# History of medical genetics in South Africa

**DOI:** 10.4102/jcmsa.v2i1.117

**Published:** 2024-09-27

**Authors:** Jennifer G. Kromberg, Grietje de Jong

**Affiliations:** 1 Division of Human Genetics, Faculty of Health Sciences, University of the Witwatersrand, Johannesburg, South Africa; 2 Independent Researcher, Stellenbosch, South Africa

Medical genetics is one of the newest of the medical specialities. It was only after the deoxyribonucleic acid (DNA) model was constructed and understood by Watson and Crick in 1953^1^ and the number of chromosomes was finally established as 46 that clinical applications of these biological sciences began to become a possibility.^2^ At that time in South Africa (SA), studies on the chromosomes were being undertaken by well-known (and later world-famous) scientists, such as Dr Sydney Brenner and Dr Philip Tobias.^3^ Cytogenetic laboratories were set up at the SA Institute for Medical Research (now National Health Laboratory Service [NHLS]) in Johannesburg in the 1960s,^4^ and at the University of Cape Town (UCT) in the early 1970s.^5^ The first Departments of Medical Genetics then developed and chairs were established at UCT in 1972 and the University of the Witwatersrand (Wits) in 1974.^3,5^ Formal genetic services focused on a broad range of services including cytogenetics, diagnosis of genetic pathology, genetic counselling and research.

The University of Stellenbosch set up a cytogenetics laboratory in 1965, which was expanded in the 1970s into a division of Medical Genetics. Later, in the 1980s, the University of Pretoria created a Department of Human Genetics, and dedicated units, staffed by paediatricians with an interest in genetics, were developed in Durban and Bloemfontein.

In 1977, the Minister of Health announced that genetic services would be integrated into the Department of Health (DoH) and financed by the state under the *Health Act* (*Act 63 of 1977*). Laboratory services were then expanded, cytogenetic testing became more widely available and prenatal diagnosis increased. Community genetics services were initiated in 1974,^6^ and genetic nurses were appointed in several regional offices to create awareness of genetic disorders and identify and support cases. By 1982, there were eight cytogenetic laboratories,^6^ mostly in academic institutions, and private laboratories were developing.

When the expertise became available, serogenetics, biochemical and molecular genetics laboratories, and a human diversity research laboratory to study the origins of southern African peoples, were established. Genetic counselling clinics, set up in the early 1970s,^7,8^ also expanded. These clinics, usually staffed by health professionals with academic appointments, were developed in all the major cities, and outreach genetic clinics were held in various peripheral centres.^5^ In 2008, for example, 7313 consultations were conducted by about 18 clinical staff in clinics across the country.^8^

In 2001, the DoH published the policy guidelines for the Management and Prevention of Genetic Disorders, Birth Defects and Disabilities. To meet the needs of the SA population, 70 medical geneticists, 300 genetic counsellors and 300 laboratory scientists were required.^4^ The provision of permanent posts by the NHLS and provincial governments increased only slightly at that time. Despite this ongoing lack of DoH support, the academic excellence developed in clinical teaching, training and research in medical genetics has been maintained over the years.^5^

Medical geneticists, genetic counsellors and medical scientists are all being trained in the country. A masters’ degree in Genetic Counselling was introduced at Wits in 1989, and at UCT in 2004, and the profession was recognised by the Health Professions Council of SA in 1992. Trained genetic counsellors now counsel about half the cases seen at genetic clinics, as well as having research and teaching roles,^9,10^ and eight have completed doctoral studies. Medical Genetics was first recognised in 1999 as a sub-speciality in Medicine, and in 2007 it became a primary speciality. Training over 4 years, completion of a research project and Master of Medicine (MMed) degree, and Fellowship of the College of Medicine are now required for specialist registration.

The main research focus, from the early years, was on investigating the epidemiology of genetic disorders occurring in local population groups. The unique features within these groups have been clarified by clinical and molecular genetics research, and founder mutations for several conditions, for example, oculocutaneous albinism and cystic fibrosis, have been identified.^11^ Funding from the SA Medical Research Council has supported many of these projects.

In the last decade, with the expansion of the African Society of Human Genetics (AfSHG), attention has been directed to genetic developments in Africa, the building of research networks and the Human Heredity and Health Africa (H3Africa) programme. Support has come from the Welcome Trust, National Institute of Health and other donors. Furthermore, the Sydney Brenner Institute for Molecular Bioscience was formally established at Wits in 2014 as an African Genomics Research hub with a focus on strengthening the continent’s capacity to analyse large epigenomic datasets for the purpose of promoting precision medicine approaches in Africa.

The World Health Organization^12^ has reported that 7.6 million children are born annually worldwide with severe genetic conditions, and about 90% of these births are in low- or middle-income countries. In these countries, the health focus is often on communicable diseases, such as human immunodeficiency virus (HIV) and/or acquired immunodeficiency syndrome (AIDS) and tuberculosis. Nevertheless, medical genetics departments in SA, although understaffed and able to meet only 10% of the need, are comparable to any in the world and provide comprehensive services targeted to the genetic needs of local populations.
